# The Molecular Mechanism of Bisphenol A (BPA) as an Endocrine Disruptor by Interacting with Nuclear Receptors: Insights from Molecular Dynamics (MD) Simulations

**DOI:** 10.1371/journal.pone.0120330

**Published:** 2015-03-23

**Authors:** Lanlan Li, Qianqian Wang, Yan Zhang, Yuzhen Niu, Xiaojun Yao, Huanxiang Liu

**Affiliations:** 1 School of Pharmacy, Lanzhou University, Lanzhou, China; 2 State Key Laboratory of Applied Organic Chemistry and Department of Chemistry, Lanzhou University, Lanzhou, China; 3 Key Lab of Preclinical Study for New Drugs of Gansu Province, Lanzhou University, Lanzhou, China; 4 State Key Laboratory of Quality Research in Chinese Medicine, Macau Institute for Applied Research in Medicine and Health, Macau University of Science and Technology, Taipa, Macau, China; Massachusetts General Hospital, UNITED STATES

## Abstract

Bisphenol A (BPA) can interact with nuclear receptors and affect the normal function of nuclear receptors in very low doses, which causes BPA to be one of the most controversial endocrine disruptors. However, the detailed molecular mechanism about how BPA interferes the normal function of nuclear receptors is still undiscovered. Herein, molecular dynamics simulations were performed to explore the detailed interaction mechanism between BPA with three typical nuclear receptors, including hERα, hERRγ and hPPARγ. The simulation results and calculated binding free energies indicate that BPA can bind to these three nuclear receptors. The binding affinities of BPA were slightly lower than that of E2 to these three receptors. The simulation results proved that the binding process was mainly driven by direct hydrogen bond and hydrophobic interactions. In addition, structural analysis suggested that BPA could interact with these nuclear receptors by mimicking the action of natural hormone and keeping the nuclear receptors in active conformations. The present work provided the structural evidence to recognize BPA as an endocrine disruptor and would be important guidance for seeking safer substitutions of BPA.

## Introduction

The item endocrine disruptor chemicals (EDCs) were firstly coined in 1991 at the Wingspread Conference Center in Wisconsin. The paper by Theo Colborn et al. in 1993 was one of the earliest papers about this phenomenon [1]. EDCs are referred to those exogenous substances that can interfere with the endocrine system and then lead to a range of developmental, reproductive, immune, neurological, or metabolic diseases in human and animals [2,3]. Many EDCs are man-made chemicals produced and are released into the environment by industry production such as plasticizers, organotins, pesticides, or alkylphenols [4]. In recent years, many efforts were made to investigate the detailed influence of EDCs on mammal systems. There are many evidences showing the hazardous effects on thyroid function, brain function, obesity and metabolism, insulin and glucose homeostasis [5].

Among the known EDCs, Bisphenol A (BPA) is one of the alkylphenol EDCs [6]. BPA has a symmetrical chemical structure of HO-C_6_H_4_-C(CH_3_)_2_-C_6_H_4_-OH and has attracted considerable attention and controversy due to its present in many environmental and human samples. It is used abundantly in the manufactory of polycarbonate plastics and epoxy resins. It is also used in a large number of plastic products of our daily life, including drinking water bottles, baby bottles, dentistry sealants, canned food and beverage packaging [7,8]. Due to the wide applications of BPA, organisms can be easily in exposure to BPA-containing environment. The main source of human exposure to BPA is likely to be through food and drinks in contact with materials containing BPA [9]. Some researches indicate that skin contact may also contribute to human exposure of BPA [10]. Additionally, some other studies show that BPA is released from consumed products, leading to its detection in food, drinking water, wastewater, air and dust [9,11]. Meanwhile, it has been hypothesized that human exposure to BPA in the early stage of development could also lead to the onset of obesity and other metabolic syndrome [12,13].

At present, many lines of evidence revealed that BPA could interact with many nuclear hormone receptors such as estrogen receptor [4,11, 14, 15–17], human estrogen-related receptor [11,18,19], human pregnane X receptor or steroid and xenobiotic receptor [20], androgen receptor [18,21], human peroxisome proliferator activated receptor [22] and thyroid hormone receptor [23,24]. Therefore, BPA could disturb the normal function of nuclear receptors. Nuclear receptors are a class of proteins within cells that are responsible for sensing hormones. Nuclear receptors are ligand-inducible transcription factors specifically regulate the expression of target genes involved in metabolism, development, and reproduction [25]. They are of major importance for intercellular signaling in animals because they transmit different intra and extracellular signals on the regulation of genetic programs. Endogenous hormones such as progestins, estrogens, androgens, glucocorticoids, vitamin D3, thyroid and retinoid hormones can activate the normal function of nuclear receptors. Meanwhile, endocrine disruptors from exogenous environment can also interact with nuclear receptors ligand binding domains (LBDs) and then give rise to disorder of downstream signaling pathways. Because nuclear receptors regulate the expression of a large number of genes, chemicals that could activate these receptors would have profound effects on the organisms. Many of these regulated genes are associated with various diseases such as cancer, osteoporosis, obesity [26,27]. Nearly 13% targets of U. S. Food and Drug Administration (FDA) approved drugs are nuclear receptors [28]. It would lead to serious consequence when the normal function of nuclear receptors was obstructed by exogenous small molecules.

Although *in vivo* or *in vitro* biological assays have shown that BPA can disturb the normal function of nuclear receptors [16,19,20,29]. The detailed molecular mechanism about how BPA interacts with nuclear receptors and affects their function is still undiscovered. As a useful computational method, molecular dynamic (MD) simulations have the ability to display the detailed and dynamics interaction features between ligands and receptors. It has been successfully used to uncover numerous ligand-receptor interaction mechanisms which are difficult to describe by experimental assays [30,31]. In this study, to disclose the mechanism how BPA interacts with nuclear receptors, all-atom molecular dynamics (MD) simulations and molecular mechanics generalized Born surface area (MM-GBSA) calculations [32–34] were performed to explore the detailed interactions of BPA with the LBDs of human estrogen receptor α (hERα), human estrogen-related receptor γ (hERRγ), and human peroxisome proliferator activated receptor γ (hPPARγ). To probe if BPA interacts with nuclear receptors by mimicking the function of its natural agonist of nuclear receptors, the interaction between hERα with estradiol (E2) was also simulated.

## Materials and Methods

### Preparation of the initial structures for molecular dynamics simulations

The atom coordinates of the complexes of hERα-BPA (PDB code: 3UU7 [11], 2.2 Å), hERα-estradiol (PDB code: 1GWR [35], 2.4 Å), hERRγ-BPA (PDB code: 2E2R [19], 1.6 Å) and hPPARγ (PDB code: 3PRG [36], 2.9 Å) were obtained from the RCSB Protein Data Bank. As there was no hPPARγ-BPA complex available, the Glide module in Schrodinger 2009 software [37–39] was applied to dock BPA into the binding pocket of hPPARγ[40,41]. As there are missing residues in 1GWR and 3PRG, Discovery Studio 2.5.5 (DS 2.5.5, Accelrys Inc., San Diego, CA) was used to add these missing residues in the loop regions. The added loops were refined in the Loop Refinement protocol in DS2.5.5.

**Fig 1:**
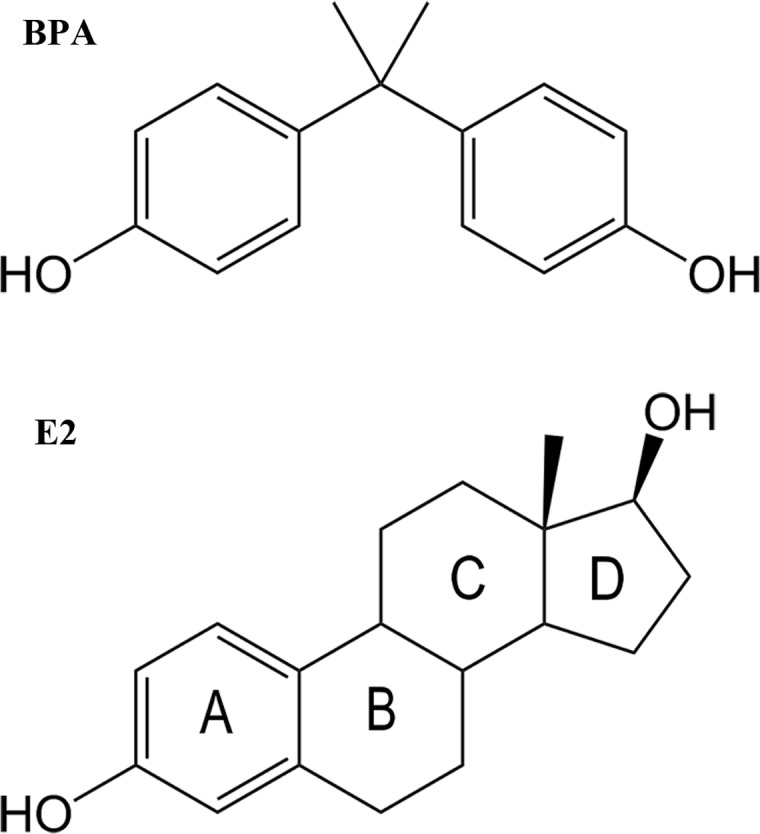
The chemical structure of the ligands. a) bisphenol A; b) 17β-estradiol (E2).

Before MD simulations, geometry optimization was performed on BPA and 17β-estradiol (E2) (Fig. 1). The electrostatic potential was calculated using Gaussian 09 program [42] at the Hartree-Fock level with 6–31G* basis set. Restrained electrostatic potential (RESP) [43–45] was generated using the antechamber module of AMBER10 to describe the partial atomic charges. The LEaP module of AMBER 10 software package was used to add all missing hydrogen atoms of the proteins. Parameters of the ligands and proteins were described by the general AMBER force field (GAFF) [46] and the standard AMBER force field (ff03) [47], respectively.

All systems were added appropriate number of sodium counter ions to keep electro-neutrality. Each system was solvated using TIP3P water [48] molecules in a octahedron box with at least 10 Å distance around the complex. As a result, the hERα-estradiol system contains 29436 atoms with 8747 waters; the hERα-BPA system contains 32548 atoms with 9515 waters, the hERRγ-BPA contains 30862 atoms with 9049 waters and the hPPARγ-BPA system contains 45521 atoms with 13649 waters. These four complexes were then used as the initial structures for the following molecular dynamics simulations.

### Molecular dynamics simulation methods

Molecular dynamics simulations were performed using the AMBER 10.0 software package [49]. For each system, three steps of energy minimization were adopted. Firstly, by restraining the protein with a force constant of 0.5 kcal/mol Å^2^, the solvent molecules and counter ions were minimized by using 2500 steps of steepest descent algorithm first and then switched to 2500 steps of conjugated gradient algorithm. Secondly, by adopting a force constant of 0.5 kcal/mol Å^2^ on the heavy atoms of the protein, the minimization was performed using the same method as the first step. Followed this step, the whole system was minimized by using 5000 steps of steepest descent method followed by 5000 steps of conjugated gradient method without any restraint. After the minimization, each system was gradually heated from 0 K to 310 K over a period of 100 ps and maintained at 310 K with a coupling coefficient of 1.0/ps. Finally, a production simulation of 100 ns was performed in the NPT ensemble at a temperature of 310 K and a pressure of 1 atm. During the simulation, periodic boundary conditions were applied and the particle-mesh Ewald (PME) method was used to handle all electrostatic interactions [50] with a cutoff of 10.0 Å using a time step of 2 fs. The SHAKE [51] algorithm was employed on all atoms covalently bonded to hydrogen atoms.

### Binding free energy calculations

Binding free energies between the studied ligands and three nuclear receptors were calculated using MM-GBSA method implemented in AMBER 10.0 software package. This method has been used successfully to binding free energy calculations [52–54]. 500 snapshots extracted from the last 15 ns MD trajectory of each system were used for MM-GBSA calculations. For each snapshot, the binding free energy is estimated as follows:

$$\Delta {\text{G}}_{\text{binding}}{\text{ = G}}_{\text{complex}}{\text{ - G}}_{\text{receptor}}{\text{ - G}}_{\text{ligand}}$$ (1)

where G_complex_, G_receptor_, and G_ligand_ are the free energy of the complex, receptor and ligand molecules, respectively. Free energy (G) was calculated based on an average over the extracted snapshots from the single MD trajectories. The free energy can be obtained from the molecular mechanics energy E_gas_, the solvation free energy G_sol_, and the solute entropy S, respectively [55].

$${\text{G = E}}_{\text{gas}}{\text{ + G}}_{\text{sol}}\text{ - TS}$$ (2)

$${\text{E}}_{\text{gas}}{\text{ = E}}_{\text{int}}{\text{ + E}}_{\text{vdw}}{\text{ + E}}_{\text{ele}}$$ (3)

$${\text{G}}_{\text{sol}}{\text{ = G}}_{\text{GB}}{\text{ + G}}_{\text{nonpolar}}$$ (4)

Where E_gas_ is the gas-phase energy; E_int_ is the internal energy; E_ele_ and E_vdw_ are the Coulomb and van der Waals energies, respectively. E_gas_ was calculated using the AMBER03 molecular mechanics force field. G_sol_ is the solvation free energy and can be decomposed into polar and nonpolar contributions. G_GB_ is the polar solvation contribution calculated by solving the GB equation. Dielectric constants for solute and solvent were set to 1 and 80, respectively. G_nonpolar_ is the nonpolar solvation contribution and was estimated by the solvent accessible surface area (SAS) determined using a water probe radius of 1.4 Å. The surface tension constant γ was set to 0.0072 kcal/mol/Å^2^ [56].

In order to obtain the detailed interaction of two ligands with nuclear receptors, MM-GBSA calculated binding free energy were decomposed to the contribution of each residue by only considering molecular mechanics energies and solvation energies without considering entropy.

## Results and Discussion

### Stability of the simulation systems

**Fig 2:**
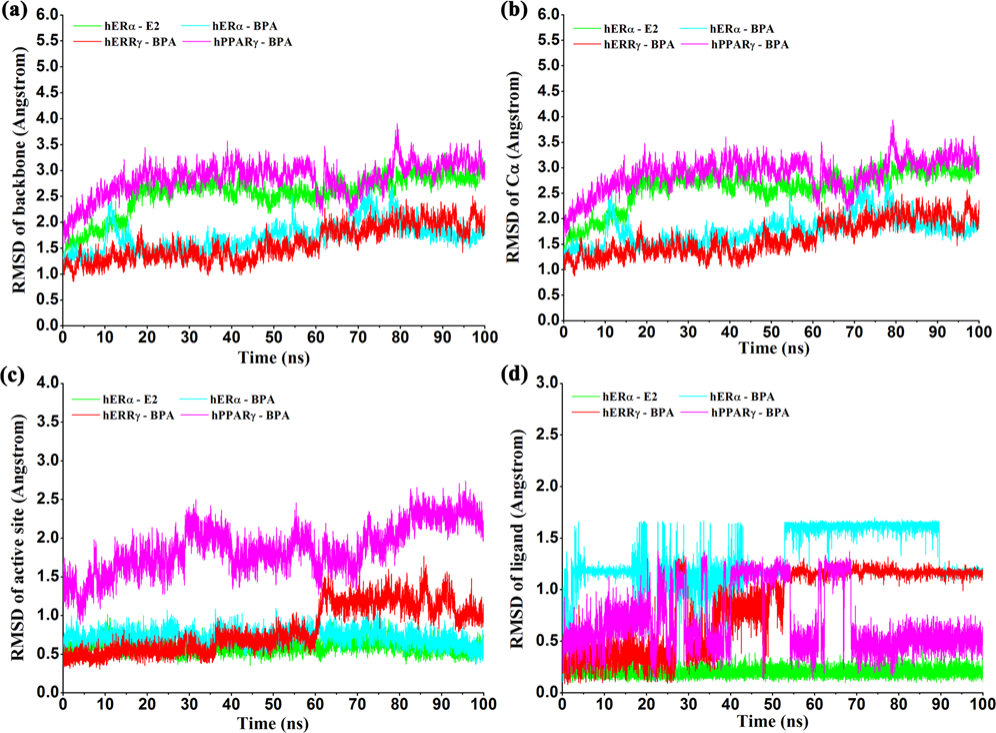
Monitoring of the equilibration of the MD trajectories of the four complexes. a) Time evolution of the RMSD of all protein backbone atoms (C, CA, N); b) Time evolution of the RMSD of Cα atoms of the protein; c) Time evolution of the RMSD of the Cα atoms of residues around 5 Å of the ligand; d) Time evolution of the RMSD of heavy atoms for the ligands.

**Fig 3:**
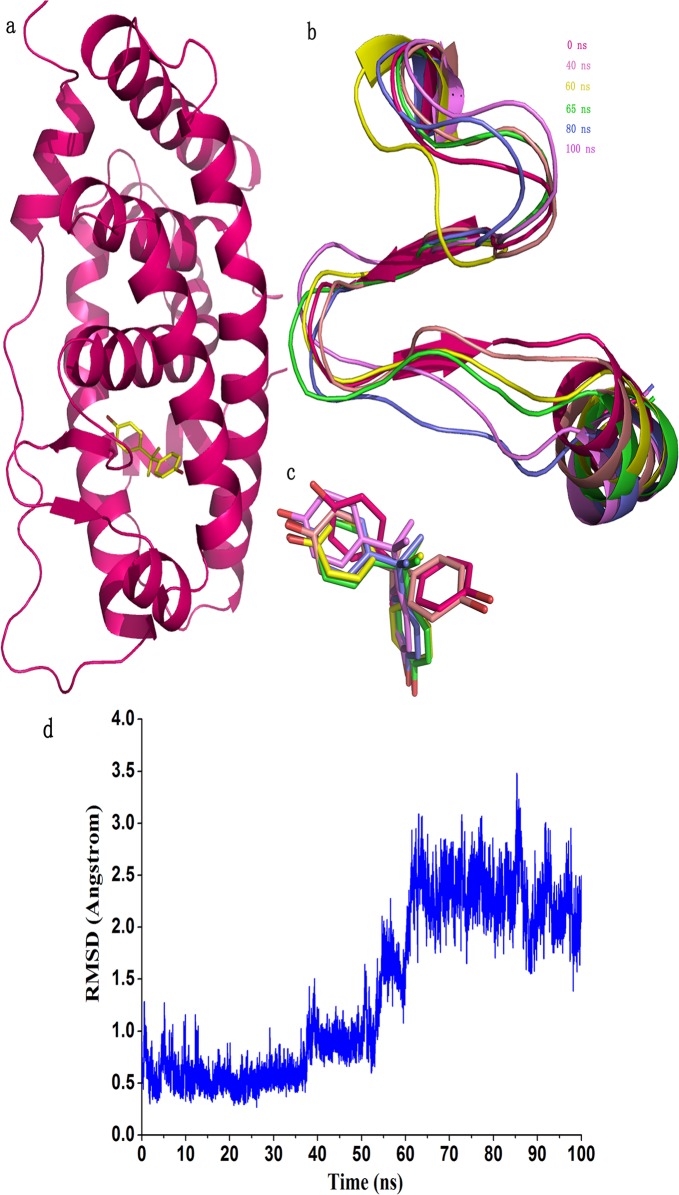
Conformation change of H6, β1 and β2 of hERRγ (residues 317–341). a) The initial structure of the protein with BPA shown in yellow sticks; b) The conformations of the studied regions at different times; c) The poses of BPA in the binding pocket at different times; d) Time revolution of the RMSD of backbone atoms of residues 317–341.

To monitor the equilibration of each system, the root-mean-square-deviation (RMSD) of protein backbone atoms, protein Cα atoms, Cα atoms of the residues within 5 Å around the ligand and the heavy atoms of BPA and E2 with respect to the initial structures were monitored and shown in Fig. 2. From Fig. 2a and 2b, it can be seen that the RMSD values of the protein backbone and Cα for hERα-BPA and hERRγ-BPA systems raised up slowly ranging from 1.0Å ~ 2.2Å and ultimately attained equilibrium at about 80ns. However, for of hPPARγ-BPA and hERα-E2 systems, the RMSD values of protein backbone increased sharply at the first 15ns to about 3.0Å and then fluctuated around 2.5Å ~ 3.5Å at the rest time, indicating the conformation change of the proteins. As shown in Fig. 2c, the RMSD values of the active sites for hERα-BPA and hERα-E2 were less than 1 Å and were stable through the simulations, indicating no significant structural fluctuations of the active sites. However, for hERRγ-BPA and hPPARγ-BPA, the evolution of RMSD values was not so stable. For the complex of hPPARγ-BPA, it is easy to understand since hPPARγ has a much larger pocket and the complex of hPPARγ-BPA was obtained by molecular docking. In the complex of hERRγ-BPA, RMSD values of the active sites were stable around 0.5~0.8 Å until the significant increase to above 1.0 Å at 60 ns, which was mainly caused by the conformational change of H6, β1, β2 and the link loops adjacent to them (residues 317–341) (Fig. 3b and 3d). By visual inspection of the conformation change, overturn of one ring of BPA to the opposite direct induced the H6 conformation change. This was in accordance with the RMSD evolution of BPA in hERRγ (Fig. 2d), which revealed the multi-conformations of BPA in the binding pocket (Fig. 3c). For the ligands (Fig. 2d), the RMSD evolution in the four complexes had quite different trends. E2 was always stable in the pocket throughout the simulation, suggesting that E2 could steadily bind with hERα. However, BPA in the three binding pockets had much larger fluctuations with quite different trends. In spite of this, BPA could achieve stable binding pose in three different pockets.

**Fig 4:**
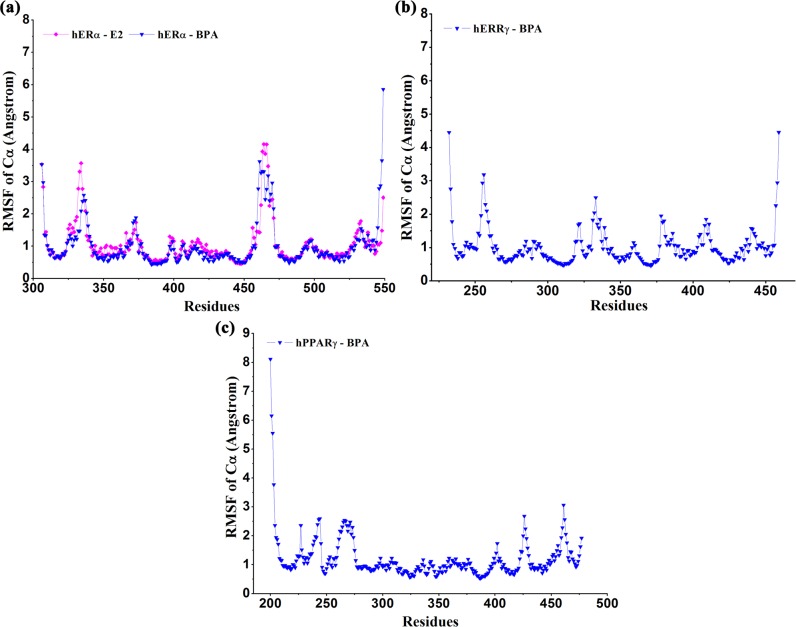
The RMSFs of Cα atoms relative to the initial structure.

In addition, the root-mean-square-fluctuations (RMSF) of Cα atoms of the proteins were also calculated and drawn in Fig. 4. As indicated in Fig. 4a, the RMSF of hERα in complex with BPA and E2 had similar values, indicating BPA and E2 bound to hERα in the same manner and interacted with hERα in a similar mechanism. When interacting with BPA, residues in hERRγ and hPPARγ (Fig. 4b and 4c) had small RMSF values. As a whole, most of the residues in four complexes were quite stable with minor mobility, except for some flexible regions that locate in the loops or two ends of proteins.

### Active conformations of the nuclear receptors

**Fig 5:**
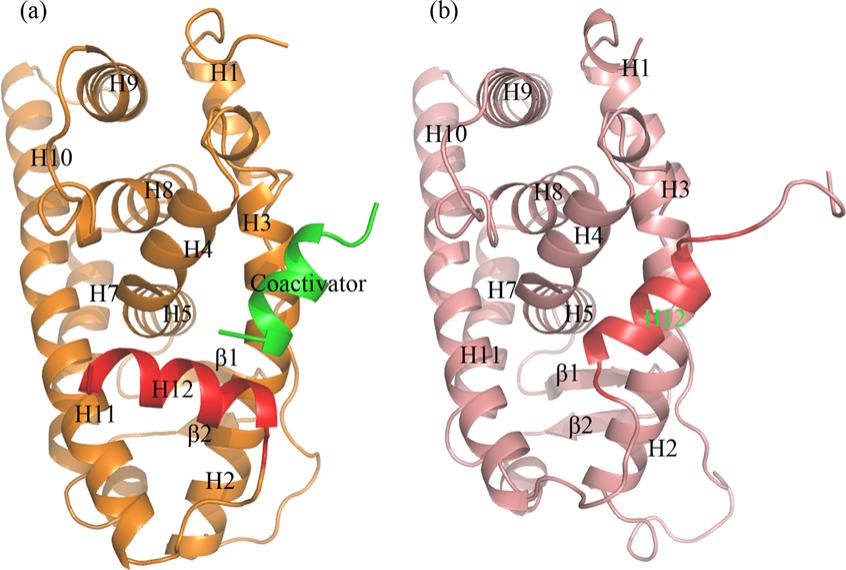
Cartoon representations of the active and inactive conformation of hERα. a) The active state of the protein with H12 in red cartoon and coactivator in green cartoon; b) The inactive state of the protein with H12 (red) covered the shallow groove which would be occupied by the coactivator.

When binding with the agonists or activators, nuclear receptors were activated by switching from inactive state (Fig. 5b) to active state with the twelfth helix (H12) sealed the hydrophobic binding pocket constituting with H2, H5, H7, H11, β1 and β2 (Fig. 5a). As an activator, E2 had the ability to stabilize hERα in the active state with H12 covering the binding pocket through the simulations and then form a shallow hydrophobic groove (helices H3–H5 and H12) for peptide coactivators binding. The receptors were then activated (Fig. 5a) and triggered downstream signaling pathways. In contrast to the active conformation, H12 was shifted away from the ligand binding pocket to occupy the shallow groove of coactivators in the inactive conformation of the receptors (Fig. 5b). When taken up by H12, coactivators could no longer bind to the groove and the downstream signaling pathway was obstructed.

The binding of BPA with hERα, hERRγ and hPPARγ could also hold H12 (As for hERRγ and hPPARγ, it refers to the helix with the same position as in hERα.) in the similar position as that in hERα-E2 complex so as to maintain the receptors activated and facilitate the binding of coactivators.

**Fig 6:**
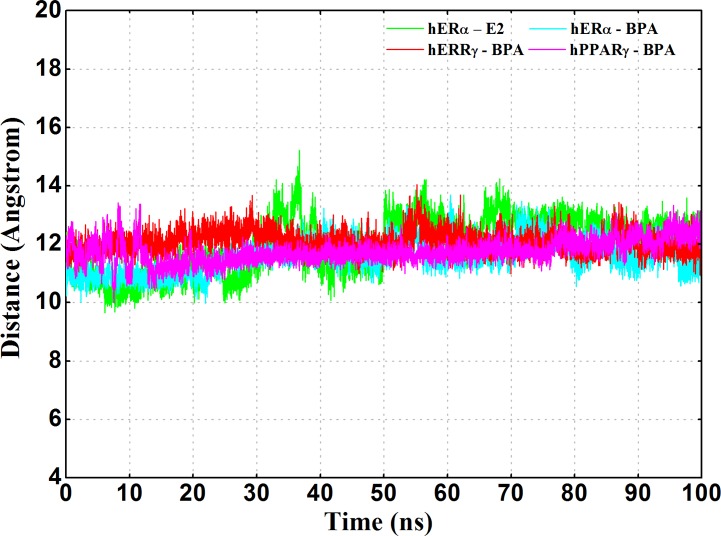
The distances between H11 and H12 of the four systems throughout the 100 ns molecular dynamics simulations.

In order to further inspect the stability of active conformations of four complexes, the distances between H12 and H11 were monitored and depicted in Fig. 6. In spite of large fluctuations at 30–40 ns of hERα-E2, there were no structural clashes and all the four complexes were able to keep the active state of proteins through the simulations with fluctuations less than 4 Å. It indicated that BPA could stably bind and interact with active nuclear receptors and keep the receptors activated.

### Binding free energy calculations for the ligands and receptors binding

**Fig 7:**
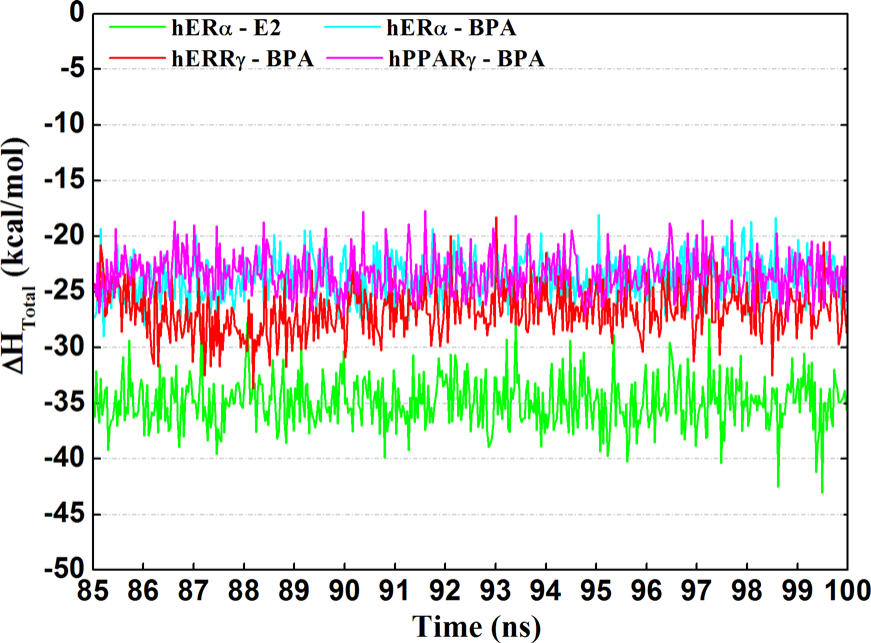
Enthalpy evolution against the last 15 ns of the four systems.

**Fig 8:**
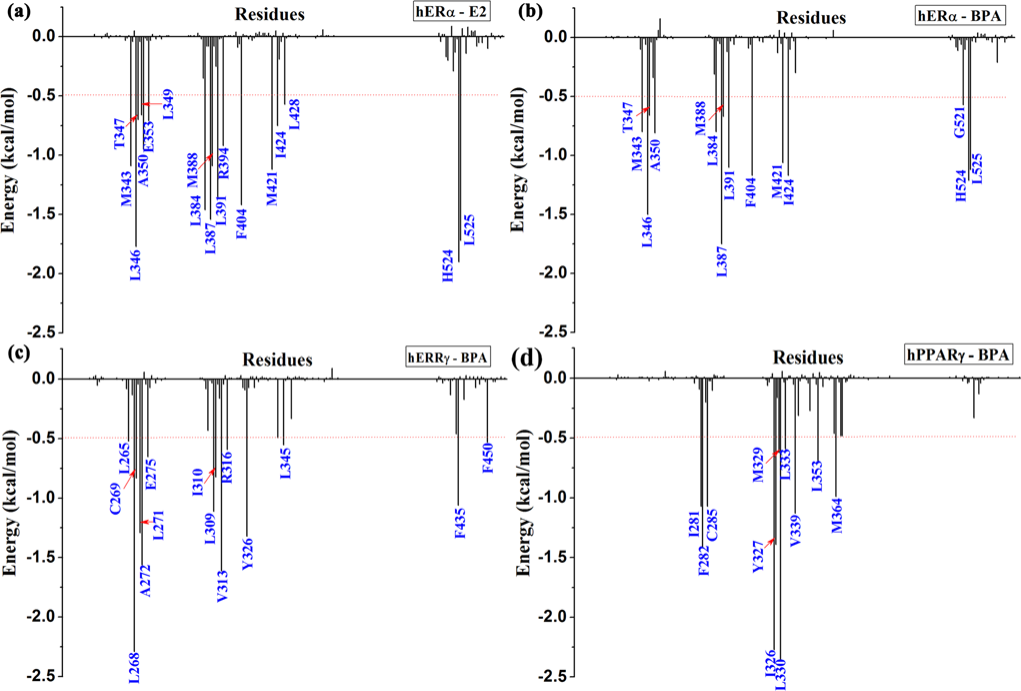
Intermolecular ligand-receptor per-residue interaction spectrum of the four complexes.

In order to gain insight into the contribution spectrum of binding energy for E2 to hERα, BPA to hERα, hERRγ and hPPARγ, the enthalpy contributions during the last 15ns of simulation were calculated for each system using the MM-GBSA method and were shown in Fig. 7. Due to long time-consuming, the entropy contributions were not calculated. The obtained binding free energies were shown in Table 1. It can be seen from Fig. 8 and Table 1, as a natural estrogen, E2 bound to the receptor with the strongest affinity with a binding free energy of -34.88 kcal/mol. Though with lower binding affinities, BPA binding to hERα, hERRγ and hPPARγ still had moderate potencies, with the binding free energies of -23.77 kcal/mol, -26.69 kcal/mol and -23.39 kcal/mol, respectively. According to the order of binding free energies, the binding potencies of BPA towards hERα, hERRγ, hPPARγ can be ranked as: hERRγ > hERα > hPPARγ, which was almost consistent with the experiment results [11,17,36]. From the contribution of various energy components of binding free energies (Table 1), the nonpolar interaction (ΔG_nonpolar_ = ΔE_vdw_ + ΔG_sol-np_) contribution is the main driving force for ligand binding. Contrary to the hydrophobic contribution, the polar interaction (ΔG_polar_ = ΔE_ele_+ ΔG_sol-ele_) contributed unfavorably to the binding of ligands. In fact, the direct intermolecular electrostatic interactions (ΔE_ele_) made favorable contribution to the binding process, while they were compensated by the large desolvation penalties (ΔG_sol-ele_) (Table 1).

**Table 1:** Calculated binding free energy and its components based on MM-GBSA method for the four systems.

Complex	Contribution(kcal/mol)								
	ΔE_ele_	ΔE_vdw_	ΔE_MM_	ΔG_sol-np_	ΔG_sol-ele_	ΔG_sol_	ΔG_polar_ ^a^	ΔG_nonpolar_ ^b^	ΔG_bind_
hERα-E2	-22.31	-41.36	-63.67	-4.96	33.75	28.79	11.44	-46.32	-34.88
hERα-BPA	-11.22	-32.88	-44.1	-4.79	25.11	20.32	13.89	-37.67	-23.77
hERRγ-BPA	-15.67	-34.9	-50.57	-4.44	28.32	23.88	12.65	-39.34	-26.69
hPPARγ-BPA	-16.02	-31.65	-47.67	-4.45	28.72	24.27	12.7	-36.1	-23.39

*Note*:

^a^ΔG_polar_ = ΔE_ele_ *+* ΔG_sol-ele_;

^b^ΔG_nonpolar_ = Δ*E*_vdw_ *+ ΔG*_sol-np_.

### Identification of the key residues responsible for the binding of BPA

**Fig 9:**
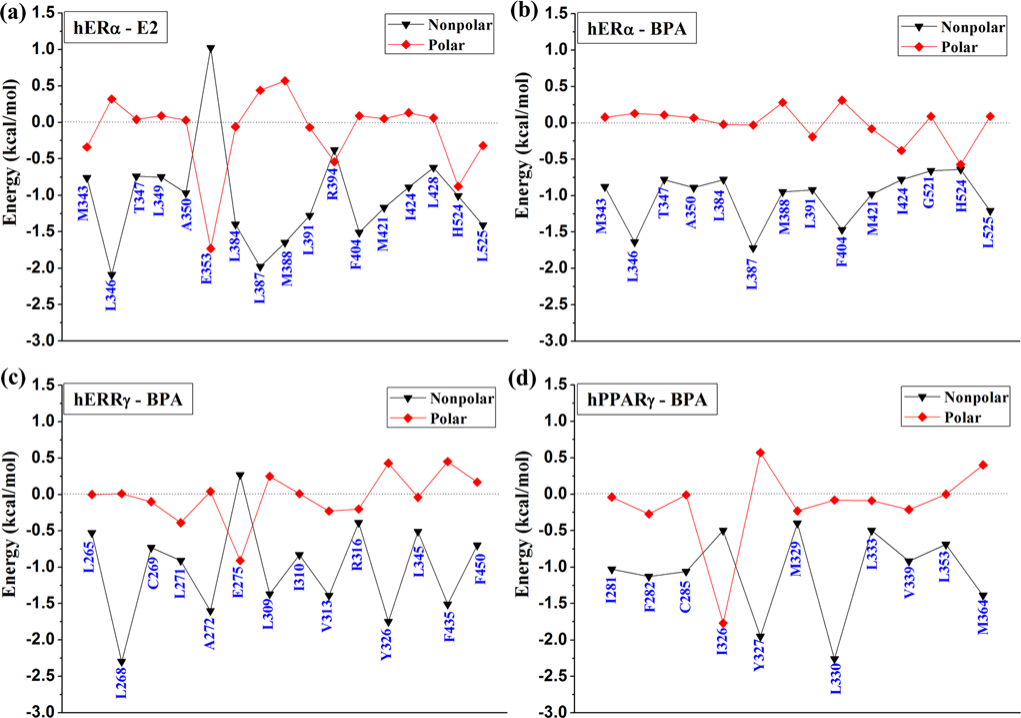
Polar and nonpolar energy contributions of the identified key residues to the complex binding.

In order to obtain a detailed profile about the binding process, the total binding free energy was further decomposed to each residue by using MM-GBSA method. The corresponding results were depicted in Fig. 8. For the binding of E2 and BPA, several key residues were identified in three receptors. For example, in hERα, the hotspot residues include M343, L346, A350, L384, L387, L391 F404, M421, H524 and L525. In hERRγ, residues L268, L271, A272, L309, V313, Y326, L345 and F435 contribute obviously to the binding of BPA. As for hPPARγ, the main hotspot residues are I281, F282, C285, I326, Y327, L330 V339, L353 and M364. The identified key residues were in well consistent with previous studies [4,11,19]. Residues with energy contribution larger than 0.5 kcal/mol were indicated for each complex in Fig. 8. The polar and nonpoalr contributions of each identified key residues were given in Fig. 9. From Fig. 7a and Fig. 7b, it can be seen that for the binding of E2 and BPA to hERα, the hotspot residues are almost the same, suggesting that BPA can interact with hERα in a similar way to E2. From Fig. 7c and Fig. 7d, the identified residues of hERRγ and hPPARγ are mostly hydrophobic. Furthermore, as shown in Fig. 8 and Fig. 9, the nonpolar interactions of a few hydrophobic residues make main contribution to the binding, indicating the hydrophobic interactions are important to the binding process of BPA to several targets. In addition, residues R394, H524 from hERα, L271, E275, V313, R316 from hERRγ and F282, I326, M329, V339 from hPPARγ also have obvious polar contributions to E2 and BPA binding.

### The comparison of binding mode of E2 and BPA to hERα

**Fig 10:**
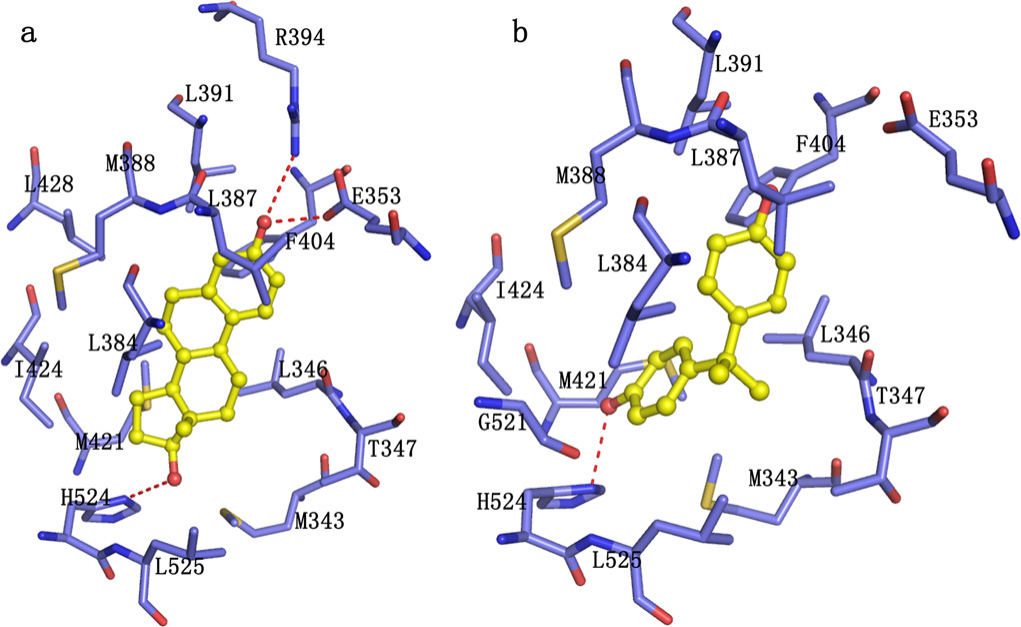
The binding pose of E2 and BPA in the binding site at 100 ns. a) Binding mode of E2; b) Binding mode of BPA. The ligands are shown in yellow ball and sticks. hERα is shown in slate cartoon. Hydrogen bonds formed between the ligand and receptors are indicated as red dashed lines.

In order to understand the detailed mechanism about how BPA affects the function of nuclear receptors, a 100 ns MD simulations of E2- hERα complex was performed as a comparison because E2 is a strong endogenous estrogen receptor agonist. In Fig. 10a, the core ring section of E2 formed strong interaction with several hydrophobic residues of hERα, such as Leu346, Leu384, Leu387, Met388, Phe404, Met421 and Leu525. The T-shape stacking between the phenyl group of E2 and the phenyl group of Phe404 could still be favorable to the ligand binding. Moreover, the hydrogen bonds formed between the ring A and Glu353 and Arg394 as well as the hydrogen bonds between the ring D and His524 further strengthened the ligand-receptor interaction throughout the simulation. Compared to E2, BPA binding to hERα adopted a fashion similar to E2 with two phenol fragments pointing to two ends of the hydrophobic pocket, one ring to residues Glu353 and the other to His524 (Fig. 10b). Even so, the hydrogen bond interactions formed between BPA and Glu353, Arg394 and His524 greatly reduced compared with the coresponding hydrogen bond interaction for E2 (Table 2). The nonpoalr component of the binding free energy contributing to BPA binding mainly came from the van der Waals contibution of residues Met343, Leu346, Leu387, Leu391, Phe404, Met421 and Leu525. However, BPA has a smaller structure than that of E2 and occupies less space in the binding pocket. Thus, the interaction between BPA and several residues was impaired to a certain degree, such as the van der Waals contribution of M343, Leu384, Met388, I424, L428 and Leu525. The electrostatic contirbution comes from Met343, Glu353, Arg394 and His524 (Fig. 8 and Fig. 9), leading that the direct interaction between BPA and hERα weakened.

**Table 2:** Occupation of H-bond between the key residues of receptor and ligand in the four systems.

Complex	Donors and Acceptors	Occupation (%)
hERα-E2	E353@OE1—E2@O1-H	72.61
	H524@ND1—E2@O2-H	51.20
	E353@OE2—E2@O1-H	15.66
hERα-BPA	E353@OE1—BPA@O2-H	31.03
	H524@ND1—BPA@O1-H	15.52
hERRγ-BPA	E275@OE2—BPA@O1-H	59.44
	E275@OE1—BPA@O1-H	11.35
	N346@OD1—BPA@O2-H	14.11
hPPARγ-BPA	I281@O—BPA@O2-H	5.01

*Note*: The hydrogen bonds were defined by acceptor∙∙∙donor atom of distances less than 3.5Å and acceptor∙∙∙H-donor angles larger than 120°.

### Structural basis of BPA binding to hERRγ and hPPARγ

**Fig 11:**
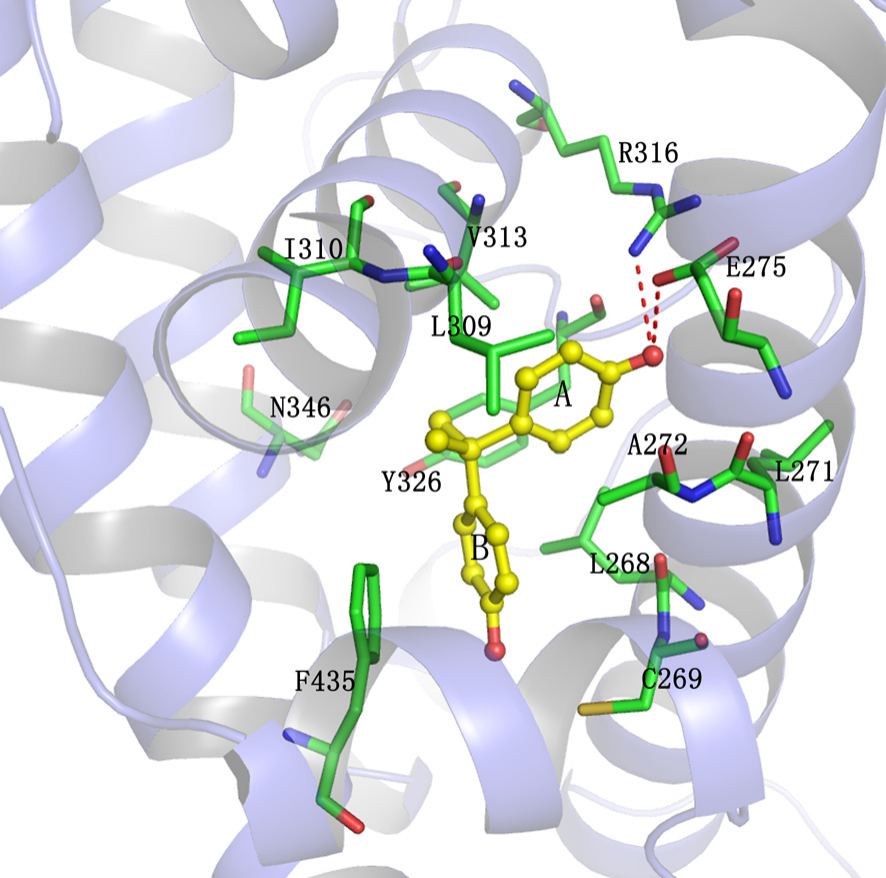
The interaction between BPA and hERRγ. Protein is shown in cartoon colored slate. Key residues are shown in green sticks. BPA is shown in yellow ball and stick. Direct hydrogen bond is indicated as red dashes.

Fig. 11 shows the direct interaction between BPA and hERRγ. As can be seen, BPA binds to hERRγ with two nonplanar phenol fragments pointing toward two ends of the binding pocket, wchi is similar to that of E2 and BPA in the pocket of hERα. In the initial structure, BPA formed hydrogen bonds interaction with Glu275, Arg316 at ring A in one end and with Asn346, Tyr326 at ring B in the other end. The hydrogen bond formed by BPA and Glu275 was monitored almost throughout the simulations (Table 2, the occupancy is about 70%). As the ring B of BPA rotated away from Asn346 and Tyr326 to a more stable position, the hydrogen bond network between these two residues were interrupted (Fig. 3c and Table 2). Herein, the hydrogen bond between BPA and Glu275 played key role in the ligand-receptor interaction. In addition, most residues of the binding pocket surrounded BPA were hydrophobic. These hydrophobic residues such as Leu268, Leu271, Ala272, Leu309, Ile310, Val313, and Phe435, form strong hydrophobic interactions with BPA. Besides, the face-to-face interaction formed between the B ring of BPA and Phe435 was also strong (Fig. 11).

**Fig 12:**
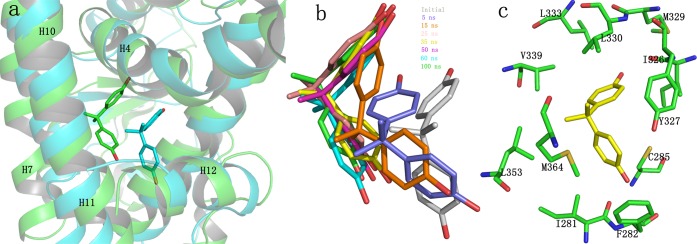
Direct interactions between BPA and hPPARγ. a) Superimposition of the initial structure (cyan) to the 100ns structure (green) of hPPARγ-BPA complex with BPA shown in sticks; b) Movement of BPA in the binding pocket at different times; c) Sticks representation of the interaction between BPA (yellow) and hPPARγ (green).

The interaction between BPA and hPPARγ was given in Fig. 12. As the initial complex structure was obtained using docking method, BPA in the binding pocket moved a lot to have a more appropriate location (Fig. 12). Although BPA is moved in the pocket, the two rings of BPA extended toward two directions, similar to that in hERα and hERRγ. BPA has van der Waals and electrostatic interactions with residues Ile281, Phe282, Cys285, Ile326, Leu330, Val339, Met364 and Tyr327 of hPPARγ(Fig. 12c). Because the buried binding pocket in hPPARγ has a volume larger than that in hERα and hERRγ [36], the direct hydrogen bond interactions were crippled. In spite of this, the van der Waals and electrostatic contributions could still favor the binding and trap BPA in the binding pocket.

Overall, the obtained results show that BPA can bind and interact stably with three nuclear receptors and keep the twelfth alpha helix sealing the hydrophobic ligand binding pocket. With the twelfth alpha helix covering the pocket, the receptors were trapped in an active conformation which allowed the binding of coactivators and would transduct the downstream gene transcription signaling. Once activated abnormally by exogenous chemicals, the normal regulation of endocrine system was disorganized and normal functions were disordered.

## Conclusions

In this study, the molecular mechanism of BPA binding to hERα, hERRγ and hPPARγ was investigated by using molecular dynamics simulations and MM-GBSA calculations. The simulation results demonstrate that BPA can bind to the hydrophobic pocket of the three studied nuclear receptors. The twelfth helix (H12) of the binding pocket was in activated state when BPA binding to receptor. BPA also has stable interaction with the nuclear receptors by mimicking the behavior of natural hormone E2. The calculated binding free energies are favorable to BPA binding and the binding process is mainly driven by van der Waals and hydrogen bond interactions. The calculated binding free energy of BPA to hERRγ is the lowest. The binding free energy to hPPARγ is the highest. Binding mode analysis suggests that BPA can stay in the pocket with two rings extended to interact with the residues in the pocket. The obtained results can provide structural evidence of BPA as an endocrine disruptor and will be great importance in the guidance of searching for safer BPA substitute.
